# Surface Roughness of Z-Cut Quartz Etched by Ammonium Bifluoride and Ammonium Bifluoride Mixed with Isopropyl Alcohol Solutions

**DOI:** 10.3390/mi8040122

**Published:** 2017-04-13

**Authors:** Zhaoyun Zhang, Wei Su, Bin Tang, Wei Xu, Zhuang Xiong

**Affiliations:** Institute of Electronic Engineering, China Academy of Engineering Physics, Mianyang 621999, China; weisu@caep.cn (W.S.); John46311@hotmail.com (B.T.); xw198877@gmail.com (W.X.); machoxiong@gmail.com (Z.X.)

**Keywords:** MEMS, wet etching, quartz, surface roughness, additive

## Abstract

It is meaningful to study the surface morphology of monocrystalline material, but there are few studies on the surface roughness of quartz. So, surface roughness of Z-cut quartz etched by pure ammonium bifluoride and ammonium bifluoride mixed with isopropyl alcohol (IPA) solution was investigated for the first time in this paper. Firstly, when etching in pure ammonium bifluoride solutions, the surface roughness change with etching time, etching temperature, and solution concentration were studied. Then, the surface roughness improvement given by isopropyl alcohol solution was analyzed carefully. The experimental results indicated that: the surface roughness of Z-cut quartz (0001) plane increased with etching time, but decreased with etching temperature and solution concentration; the adding of isopropyl alcohol in ammonium bifluoride solution could decrease the roughness and improve the surface quality. This is the first systemic research of the evolution of quartz surface roughness when etching in ammonium bifluoride solution, and will benefit the future design and manufacture of quartz MEMS devices.

## 1. Introduction

There are two reasons for the longstanding lack of interest in quartz. One essential reason is that the amount of knowledge and manufacturing equipment for quartz is neither as extensive, nor as widespread, as for semiconducting materials such as Si and GaAs. Another reason is the small size of commercially used quartz wafers, which reduces the cost efficiency for the industrial production of sensors. However, quartz has excellent properties, such as outstanding piezoelectric properties, low temperature coefficient, offers good electric insulation, and allows optical transmission in the wavelength range of UV light. These features have favored the utilization of quartz as a substrate for the fabrication of several types of MEMS, such as resonators [1], tuning-fork probes [2] or microlenses [3], particularly in the field of vibrating inertial sensors—i.e., vibrating beam accelerometer (VBA) [4,5,6,7] and Coriolis vibrating gyro (CVG) [7,8,9,10,11], based on a resonator as sensitive element.

In the case of the micromachining of quartz-based MEMS, wet etching is one of the most widely used methods, benefiting from similar features as silicon etching: low cost, the creation of flat surfaces, and the possibility of batch processing. Because of the complex anisotropic wet etching and sidewall arrises in fabrication of micromechanical devices, it is difficult to predict the result of quartz etching [12], so there have been many studies focusing on the simulation of the etched profile [13,14,15]. Hedlund et al. [16] studied the etching properties of Z-cut quartz in hydrofluoride-based etchants; the side-wall slopes varied with different orientation, etchants, and temperatures, the anisotropy and etching rate increased with HF concentration, and the transition between various planes can be described by an interaction of adjacent natural crystal planes. Danel et al. [17] realized an acceleration sensor and described the chemical etching of quartz crystals. They gave the etch rates of *X*, *Y*, and *Z* direction, but the etch rates of sidewall planes were not provided. Liang et al. [18] etched a quartz wafer with saturated ammonium bifluoride (NH_4_HF_2_) solution at 87 °C, and reported wet etching profile and etch rates of the etching planes which appeared. Wang. et al. [19] studied the sidewall arris flatting process in quartz fabrication etching in hydrofluoride-based etchants. In conclusion, these studies were mainly focused on the etching properties, such as etched profiles and etching rate in different etchants, such as HF, HF+NH_4_F, NH_4_HF_2_. However, the surface roughening and the methods to improve the surface quality have not been studied.

There are many studies on etched single-crystal silicon surface [20,21], but few on the roughness of the etched quartz surface. There are two important reasons for studying the surface morphology of monocrystalline material. The first is that the surface morphology of monocrystalline material can help us to understand the anisotropic etching mechanism. The microscopic facet structures that densely cover an etched surface seem strongly related to the anisotropic etching mechanism. Detailed information on the surface morphology allows us to understand the mechanism. The second reason is that the surface morphology of micromachined devices influences their performance; for example, the roughness of the etched surface influences the intrinsic thermal behavior of the vibrating beam accelerometer [5]. So, surface roughening was studied in this paper.

When using common etching solutions (HF, HF+NH_4_F, NH_4_HF_2_), the etched surface of quartz tends to be covered with hillocks and form a rough surface. This may be detrimental to applications. There are not many effective methods to improve the etched quartz surface quality to get mechanically reliable devices. Many additives, including isopropyl alcohol (IPA) [22], triton X-100 [23,24], and other ion-typed surfactants [25] have been reported to be able to reduce the undercut and improve the roughness of etched surface in silicon wet etching. Inspired by these results, the effect of adding additive on surface roughness in quartz wet etching should be investigated. IPA has been proven to effectively decrease the surface roughness of silicon [22,26]. So, the effects of IPA additives on the etched surface roughness of quartz was investigated in this paper.

Z-cut quartz wafers show the most potential for the fabrication of micro-sensors and micro-actuators due to the high etch rate at the *Z* plane. Device and process designers are mainly concerned with the surface quality in its (0001) plane. So, in this paper, we described our investigation of the (0001) plane surface roughness of a single-crystal quartz specimen etched with ammonium bifluoride solution. The surface roughness change with etching time, etching temperature, and solution concentration were studied. Then, the improvement given by isopropyl alcohol additive was analyzed carefully.

## 2. Experimental Method

**Figure 1:**
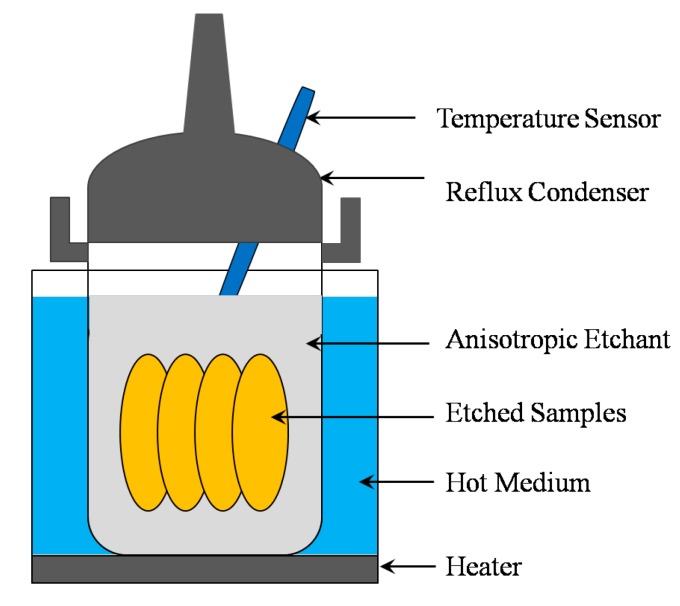
The graphical representation of the experimental setup.

A 4-inch 500 μm-thick Z-cut α-quartz wafer was used to carry out the experiments. The wafer was manually cleaved into dozens of small pieces about 10 mm × 10 mm in dimension for further experiments. Before the experiments, the samples were cleaned by using H_2_SO_4_:H_2_O_2_ = 4:1 solutions and then rinsed with DI water for a few minutes. The etchants used herein were ammonium bifluoride and ammonium bifluoride with isopropyl alcohol solutions. The ammonium bifluoride solutions were prepared by dissolving appropriate amounts of the NH_4_HF_2_ chemical into warm deionized water according to the experiments. Two series of the etching process were carried out. Firstly, the etching in the pure ammonium bifluoride solutions was carried out. In the second series, the etching in 67.8 wt % NH_4_HF_2_+IPA was studied. IPA was successively added to the NH_4_HF_2_ solution in amounts ranging from 5% (*v*/*v*) to 15% (*v*/*v*) after every etching cycle. The etching process was performed in a cylindrical polytetrafluoroethylene barrel; the graphical representation of the experimental setup is shown in Figure 1. During etching, the temperature of the solution was at 90 ± 1 °C. No etchant was added during the etching to compensate for the consumption of etchant, and no stirring of the etchant was used. The morphology of the etched surface was observed using scanning electron microscopes (SEM), and a surface profilometer (Alpha-step IQ) was used to evaluate the mean roughness (Ra).

## 3. Results and Discussion

### 3.1. Roughness Change with Etching Time

**Figure 2:**
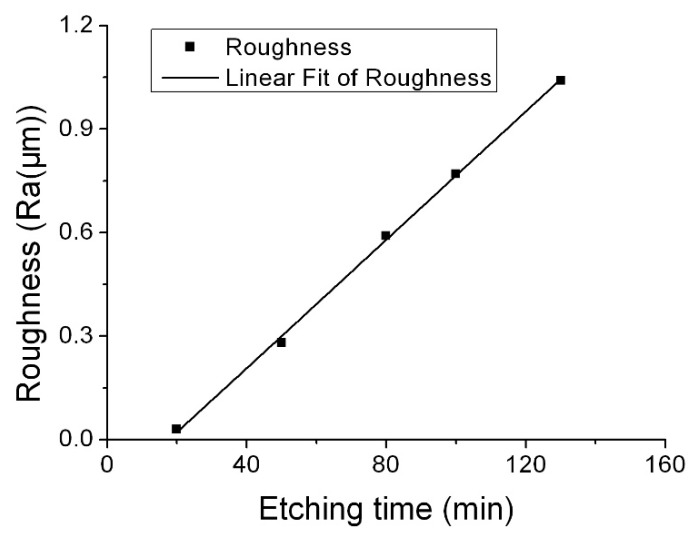
Roughness change as a function of etching time (etchant: 55.0 wt % NH_4_HF_2_, etching temperature: 90 °C).

Surface roughness change as etching time was firstly investigated. Corrosion condition: etchants 55.0 wt % NH_4_HF_2_, etching temperature 90 °C. The etching rate was 1.47 μm/min. We chose the maximum etching time (130 min) that produced an etching depth of 190 μm for the (0001) plane. Surface roughness was measured at five intervals: 20, 50, 80, 100, and 130 min. The relationship between etching time and surface roughness for the *Z* orientation is shown in Figure 2. The etched surface roughness increased with the etching time, almost in a linear relation. When the etching depth was 190 μm, the roughness value was 1.04 μm in Ra—almost 1000 times the initial roughness (<1 nm).

**Figure 3:**
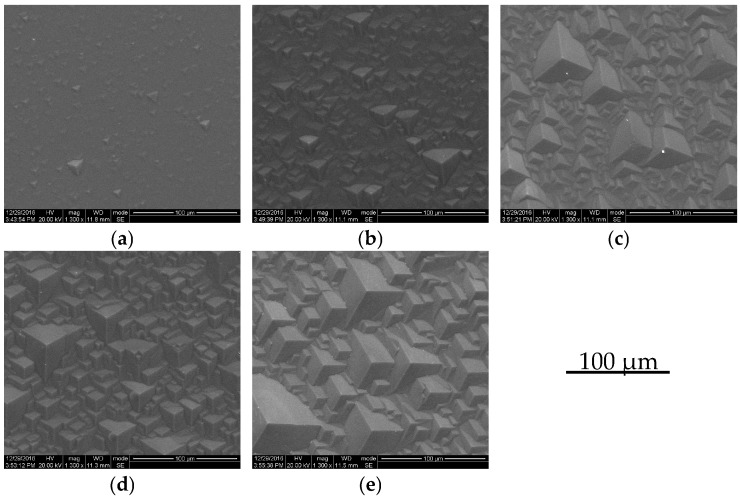
Morphology changes as a function of etching time. Etching time: (**a**) 20 min; (**b**) 50 min; (**c**) 80 min; (**d**) 100 min; (**e**) 130 min, etchant: 55.0 wt % NH_4_HF_2_, etching temperature: 90 °C.

We observed the surface morphology of the quartz (0001) plane. The morphology changed with the etching time. Photographs of the surface were taken five times during the etching, and are shown in Figure 3. The texture changed in the following manner: some hillocks (pyramids composed of three facets) gradually appeared on the surface; at the etching time of 20 min, there were a few hillocks on the surface with diameters ranging from 0 to 18 μm (height 0–1 μm), as shown in Figure 3a. At the etching time from 50 to 130 min, the number of hillocks increased, the size of hillocks grew, the maximum diameter grew from 40 to 75 μm, and the maximum height increased from 2 to 12 μm; after the etching time of 80 min, the entire surface was nearly covered by hillocks, as shown in Figure 3b–e. So, the reason that roughness increased with etching time was mainly the appearance of pyramid hillocks, which grew in number and in size with etching time.

There are few studies focused on analyzing the formation of hillocks in quartz. From the wet etching of silicon, we could deduce that the formation of hillocks on the quartz (0001) plane was mainly micromasking caused by the heterogeneity in the solution. The formation of hillocks requires four conditions to be satisfied simultaneously: (1) the existence of a micromasking caused by material impurities, bubbles formed by gas resultant, or by chemical etch residues; (2) a fast downward motion of the floor surface; (3) stable pyramidal edges; and (4) very stable pyramidal facets [27]. Conditions (2)–(4) could be simplified as V_sidewall_ < V_substrate_. For example, in the wet etching of silicon, when the solution concentration is low, the etching rate of *v*(100) > *v*(110)—satisfying the condition of V_sidewall_ < V_substrate_—many hillocks appear on the surface of the (100) plane; increasing the solution concentration, when *v*(100) < *v*(110), the condition of V_sidewall_ < V_substrate_ is not satisfied, and the hillocks on the surface of (100) plane disappear. 

In the wet etching of Z-cut quartz, the etching rate of the (0001) plane was the fastest; perpendicular to this surface were the slow-etching crystallographic m planes, V_sidewall_ < V_substrate_. So, the formation of hillocks on the quartz (0001) plane could not be avoided. The condition of V_sidewall_ < V_substrate_ could not be changed easily in the wet etching of quartz due to the crystal structure. So, eliminating the heterogeneities could be very beneficial for decreasing the number of hillocks and improving the etched surface quality.

### 3.2. Effect of NH_4_HF_2_ Concentration

We investigated the effect of NH_4_HF_2_ concentration on surface roughness. The solution concentrations were: 50.0, 55.0, 60.0, and 67.8 wt % NH_4_HF_2_ (saturated NH_4_HF_2_).

**Figure 4:**
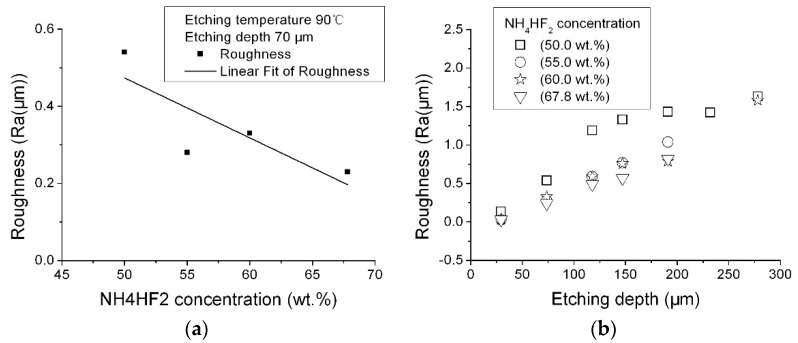
The effect of NH_4_HF_2_ concentration on roughness: (**a**) roughness change as a function of solution concentration at the same etching depth; (**b**) roughness change as a function of etching depth in different NH_4_HF_2_ concentration (etching temperature: 90 °C).

The quartz etching rates in the four solutions were 1.10, 1.47, 1.54, and 1.80 μm/min. Figure 4 shows the effect of the NH_4_HF_2_ concentration on surface roughness. As shown in Figure 1, the roughness values changed with etching time. This means that the roughness values also changed with etching depth. When we changed the NH_4_HF_2_ concentration, the etching depth changed even if the etching time was the same, because the etching rate changed as the NH_4_HF_2_ concentration changed. Therefore, we used the etching depth in the graph in Figure 3 to compare the results obtained at different NH_4_HF_2_ concentrations.

Figure 4 a shows the roughness change as a function of solution concentration at the same etching depth, Figure 4b shows the roughness change as a function of etching depth in different NH_4_HF_2_ concentration. The surface roughness of the (0001) plane increased along with etching depth for the four different NH_4_HF_2_ concentrations. In general, the surface roughness etched in the 50.0 wt % NH_4_HF_2_ solution was higher than the others, and was the lowest when etched in the saturated NH_4_HF_2_ solution. This may indicate that increasing the NH_4_HF_2_ concentration could decrease the etching surface roughness. That may be why saturated NH_4_HF_2_ solution is used in many studies.

### 3.3. Effect of Etching Temperature

**Figure 5:**
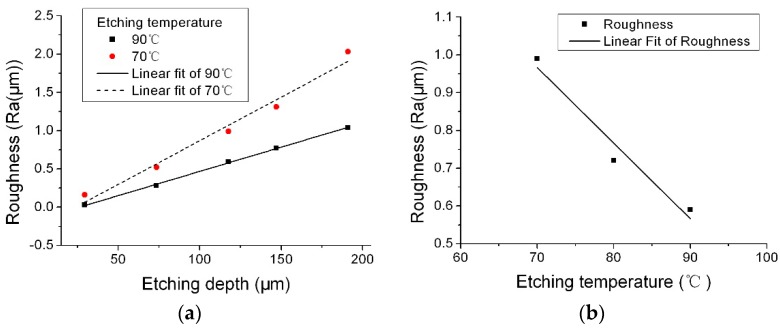
Effect of etching temperature on roughness: (**a**) roughness change as a function of etching temperature at the same etching depth; (**b**) roughness change as a function of etching depth at different etching temperature (etchant: 55.0 wt % NH_4_HF_2_).

Figure 5 shows the effect of etching temperature on roughness when the NH_4_HF_2_ concentration was 55.0 wt %. The etching depth was 70 μm. The surface roughness on the (0001) plane simply increased with the decreasing of the etching temperature and increased along with etching depth for both etching temperature of 70 and 90 °C. So, increasing the etching temperature could decrease the surface roughness. By increasing the etching temperature, the removal of reaction products could be faster, the micromasking caused by heterogeneities in reaction products could be reduced, and so the roughness could be improved by increasing the etching temperature.

### 3.4. Effect of Isopropyl Alcohol

**Figure 6:**
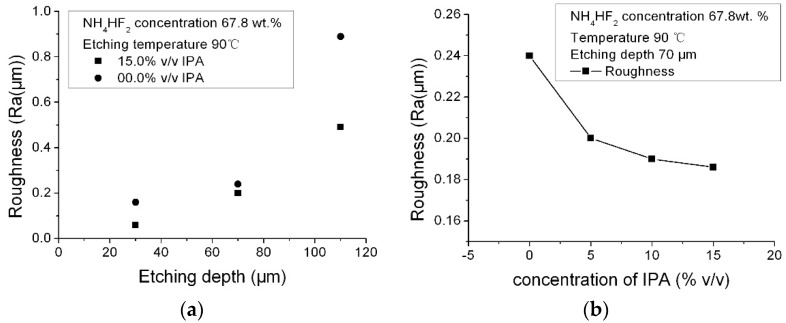
Effect of adding isopropyl alcohol (IPA) on roughness: (**a**) roughness change as a function of IPA concentration at the same etching depth; (**b**) roughness change as a function of etching depth with and without adding of IPA in the solution (etching temperature: 90°C).

The effect of adding isopropyl alcohol on roughness was investigated. The NH_4_HF_2_ solution was 67.8 wt %, IPA was successively added to the NH_4_HF_2_ solution in amounts ranging from 5% (*v*/*v*) to 15% (*v*/*v*) after every etching cycle. The etching depth was 70 μm. The results are shown in Figure 6; from Figure 6a, the surface roughness decreased along with the increase of IPA concentration. Figure 6b shows the roughness change as a function of etching depth with and without adding IPA in the solution, the IPA was effective on reducing the surface roughness along with the etching depth. 

**Figure 7:**
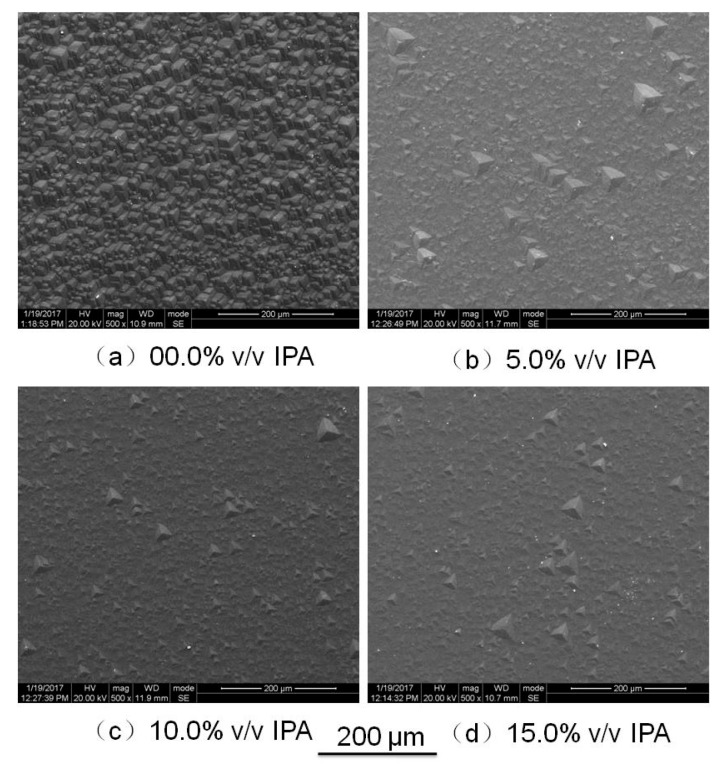
Effect of adding isopropyl alcohol on surface texture (etching temperature: 90 °C).

Figure 7 shows the effect of IPA on the surface texture. The surface of the (0001) plane etched in pure saturated NH_4_HF_2_ solution was completely covered by hillocks; when adding some IPA to the solution, the amount of hillocks decreased, and a gradual improvement could be observed in the morphology of the (0001) plane as the amount of IPA increased. However, the hillocks could not be eliminated by adding IPA. 

During the wet etching, the step bunching and micro-masking are the main reasons influencing the surface roughness. Step bunching is the result of a diffusion mechanism. Due to the transport delay and the localized consumption of the reactants at the steps, the etchant in contact with the surface develops inhomogenous regions in the proximity of the active regions. The number and size of the inhomogenous regions affect the surface roughness. By adding the IPA in the solution, the surface tension of the solution decreased. Reducing the surface tension, the transport rate of the reactants and/or products to/from the locations where they were consumed/produced increased, so the inhomogenous regions could be decreased and the etched surface roughness reduced. In addition, decreasing the surface tension could decrease the detach time of reaction products from etching surface, so could decrease the micromasking formed by chemical etching residues and improve etched surface quality.

## 4. Conclusions 

Surface roughness of Z-cut quartz etched by ammonium bifluoride and ammonium bifluoride mixed with isopropyl alcohol solution was investigated in this paper. When etching in pure ammonium bifluoride solutions, the surface roughness change with etching time, etching temperature, and solution concentration were studied. The roughness increased with the increase of etching time, and decreased along with increasing concentration and temperature of the solution. The number and size of the hillocks—which changed with etching time, etchant concentration, and etching temperature—affected the roughness of the (0001) plane. 

One method to improve the etching surface quality by adding IPA additives into ammonium bifluoride solution was proposed. By adding isopropyl alcohol in ammonium bifluoride solution, the number of hillocks reduced and the surface quality improved. This paper systemically studied the evolution of quartz surface roughness when etching in ammonium bifluoride solution, and proposed an effective method to decrease surface roughness. It will benefit the future design and manufacture of quartz MEMS devices.
